# Correlation-Based Ensemble Feature Selection Using Bioinspired Algorithms and Classification Using Backpropagation Neural Network

**DOI:** 10.1155/2019/7398307

**Published:** 2019-09-23

**Authors:** V. R. Elgin Christo, H. Khanna Nehemiah, B. Minu, A. Kannan

**Affiliations:** ^1^Research Scholar, Ramanujan Computing Centre, College of Engineering Guindy, Anna University, Chennai 600025, Tamil Nadu, India; ^2^Professor, Ramanujan Computing Centre, College of Engineering Guindy, Anna University, Chennai 600025, Tamil Nadu, India; ^3^Alumna, Ramanujan Computing Centre, College of Engineering Guindy, Anna University, Chennai 600025, Tamil Nadu, India; ^4^Senior Professor, School of Computer Science and Engineering, Vellore Institute of Technology, Vellore 632014, Tamil Nadu, India

## Abstract

A framework for clinical diagnosis which uses bioinspired algorithms for feature selection and gradient descendant backpropagation neural network for classification has been designed and implemented. The clinical data are subjected to data preprocessing, feature selection, and classification. Hot deck imputation has been used for handling missing values and min-max normalization is used for data transformation. Wrapper approach that employs bioinspired algorithms, namely, Differential Evolution, Lion Optimization, and Glowworm Swarm Optimization with accuracy of AdaBoostSVM classifier as fitness function has been used for feature selection. Each bioinspired algorithm selects a subset of features yielding three feature subsets. Correlation-based ensemble feature selection is performed to select the optimal features from the three feature subsets. The optimal features selected through correlation-based ensemble feature selection are used to train a gradient descendant backpropagation neural network. Ten-fold cross-validation technique has been used to train and test the performance of the classifier. Hepatitis dataset and Wisconsin Diagnostic Breast Cancer (WDBC) dataset from University of California Irvine (UCI) Machine Learning repository have been used to evaluate the classification accuracy. An accuracy of 98.47% is obtained for Wisconsin Diagnostic Breast Cancer dataset, and 95.51% is obtained for Hepatitis dataset. The proposed framework can be tailored to develop clinical decision-making systems for any health disorders to assist physicians in clinical diagnosis.

## 1. Introduction

Knowledge discovery plays a vital role in extracting knowledge from clinical databases. Data mining is a step in the process of knowledge discovery. The quality of data for data mining is improved using preprocessing techniques. Data mining tasks include association rule mining, classification, and clustering [1]. Data mining techniques find tremendous applications in healthcare to analyse the trends in patient records which lead to improvement in healthcare applications. Predictive data mining (PDM) plays a major role in healthcare. The goal of PDM in healthcare is to build models from electronic health records that use patient specific data to predict the outcome of interest and support clinicians in decision-making. PDM can be used to build models for prognosis, diagnosis, and treatment planning [2]. The symptoms observed on a patient, clinical examination, and outcomes of laboratory tests might perhaps exemplify more than one possible disease. Decision-making with complete certainty is not practical since there exists uncertainty in clinical data provided by the patients, and taking an accurate decision is a challenging task. PDM techniques can be applied to the data available in electronic health records to infer clinical recommendations for patients, with the aid of historic data about the clinical decisions administered to patients who exhibited similar symptoms. Computer-aided diagnosis (CAD) systems can be used by clinicians as a second opinion in decision-making and treatment planning.

A framework for knowledge mining from clinical datasets using rough sets for feature selection and classification using backpropagation neural network has been proposed in [3]. A decision support system for diagnosis of Urticaria is presented in [4]. A CAD system for predicting the risk of cardiovascular diseases using fuzzy neurogenetic approach is proposed in [5]. CAD frameworks for diagnosis of lung disorders are proposed in [6–12]. A framework for diagnosing the severity of gait disturbances for patients affected with Parkinson's disease is discussed in [13]. Classifying clinical time series data observed at irregular intervals using a biostatistical mining approach is proposed in [14]. A CAD system to diagnose gestational diabetes mellitus is presented in [15].

Classification plays a major role in CAD systems. First, the classifier is trained using a supervised learning algorithm with a train set, and second, the performance of the developed classifier is evaluated using a test set. Classification using decision tree induction, Bayesian classification, classification by backpropagation, support vector machines, and *k*-nearest neighbour classifiers are the widely used classifiers. Presence of irrelevant features in the train set affects the performance of the classifier. Pruning the irrelevant features and selecting the subset of relevant features will improve the performance of the classifier.

Feature selection algorithms can be categorized into supervised [16], unsupervised [17], and semisupervised feature selection [18] according to whether the training set is labelled or not. Filter, wrapper and embedded are supervised feature selection methods. Filter approaches to feature selection are independent of the classification algorithm used. The dependency of each and every feature to the class label is measured, and a predefined number of features are selected. Relief, Fisher score, information gain, chi-squared test, and correlation coefficient are some of the feature selection criteria that can be used in the filter approach. The wrapper method uses the predictive accuracy of a predetermined learning algorithm to determine the quality of the selected features. The embedded method first incorporates the statistical criteria, as filter model does, to select several candidate features subsets with a given cardinality. Second, it chooses the subset with the highest classification accuracy [19]. While unsupervised feature selection works with unlabelled data, it is difficult to evaluate the relevance of features. Semisupervised feature selection makes use of both labelled and unlabelled data to estimate feature relevance [20].

Computational algorithms inspired by biological processes and evolution can provide an enhanced basis for problem-solving and decision-making [21]. A review of bioinspired algorithms, namely, neural networks, genetic algorithm, ant colony optimization, particle swarm, artificial bee colony, cuckoo search, firefly, bacterial foraging, leaping frog, bat algorithm, flower pollination, and artificial plant optimization algorithm has been presented in [22]. Other bioinspired algorithms have also been proposed by researchers.

In this research work, a framework for clinical diagnosis which uses bioinspired algorithms for feature selection and gradient descendant backpropagation neural network for classification has been designed and implemented.

The rest of the paper is organized as follows.  Section 2 provides an overview of related research work. An outline of Wisconsin Diagnostic Breast Cancer (WDBC) dataset and Hepatitis dataset from University of California Irvine Machine Learning repository is presented in Section 3. Section 3 also presents a detailed description of the system framework. Results are discussed in Section 4. Conclusion and the scope for future work are discussed in Section 5.

## 2. Related Work

Leema et al. [23], in their work, developed a CAD system using a backpropagation neural network for classifying clinical datasets. Differential evolution with global information (DEGI) for global search and backpropagation (BP) for local search were used to adjust the weights of the neural network. DEGI was modelled by considering PSO's search ability and differential evolution's mutation operation that can assist in the improvement of exploration in PSO. The classifier obtained accuracies are 85.71%, 98.52%, and 86.66 when experimented with Pima Indian Diabetes dataset, Wisconsin Diagnostic Breast Cancer dataset, and Cleveland Heart Disease dataset from UCI machine learning repository, respectively.

Sweetlin et al. [24] proposed a CAD system for diagnosing bronchitis from lung CT images. The ROIs were extracted from training CT slices and from ROIs, 22 texture features in four orientations, namely, 0°, 45°, 90°, and 135°, and 12 geometric features were extracted for feature selection. A hybrid feature selection approach based on ant colony optimization (ACO) with cosine similarity and support vector machine (SVM) classifier was used to select relevant features. The training and testing datasets used in building the classifier model were disjoint and contained 200 CT slices affected with bronchitis, 50 normal slices, and 300 slices with cancer. Out of 100 features extracted from each CT slice, a subset of 60 features was selected for classification. The SVM classifier was used for classifying the CT slices. Accuracy of 81.66% with the values of *n*-max and *n*-tandem as 60 and 12 was reported.

Emary et al. [25] proposed a feature selection method using Binary Grey Wolf Optimization. Two approaches for Grey Wolf Optimization are used in the feature selection process. The objective was to maximize the classification accuracy and minimize the number of selected features. Experiments were carried out on 18 datasets from the UCI machine learning repository among which Wisconsin Diagnostic Breast Cancer dataset and lymbhography belong to clinical data. Mean fitness function values of 0.027 and 0.151 were obtained for the breast cancer and lymbhography datasets, respectively, which were comparatively greater than the values obtained using particle swarm optimization and genetic algorithm.

Nahato et al. [26] proposed a classification framework by combining the merits of fuzzy sets and extreme learning machine. Clinical datasets were transformed into fuzzy sets by using trapezoidal member function. Classification was performed using a feedforward neural network with a single hidden layer using extreme learning machine. Experiments were carried out on Cleveland heart disease (CHD) with five class labels, Cleveland heart disease (CHD) with two class labels, Statlog heart disease (SHD), and Pima Indian Diabetes (PID) datasets from UCI machine learning repository and reported accuracies of 73.77%, 93.55%, 94.44%, and 92.54%, respectively.

Mafarja et al. [27] presented a metaheuristic algorithm using Ant-Lion Optimizer for feature selection. Six variants of Ant-Lion Optimizer were analysed by deploying different transfer functions. Each transfer function was used to map the continuous search space to a discrete search space of the domain. Three V-shaped and three S-shaped transfer functions were used in this study. The experiments were conducted using 18 datasets from UCI machine learning repository and compared with PSO gravitational search algorithm and two different variants of Ant-Lion Optimizer-Based Algorithm. The experimental results show a better accuracy compared to the existing methods. For the Wisconsin diagnostic breast cancer dataset, Ant-Lion Optimizer-Based Algorithm with V-shaped transfer function obtained an accuracy of 97.4%. Ant-Lion Optimizer with V-shaped transfer function performs better than using S-shaped transfer function by avoiding local optima.

Zawabaa et al. [28] have presented a hybrid bioinspired heuristic algorithm which combines Ant-Lion Optimization (ALO) and Grey Wolf Optimization (GWO) algorithms for feature selection. In the hybrid algorithm, the convergence was obtained towards global optimization by avoiding local optima and speeding up the search process. This hybrid algorithm individually outperforms the Ant-Lion Optimizer and Grey Wolf Optimizer, which has been experimented using 18 datasets from UCI machine learning repository among which Cleveland Heart dataset and Wisconsin Diagnostic Breast Cancer dataset belong to clinical domain. The ALO-GWO algorithm showed the exploration of the search space and exploitation of optimal solution in a much balanced way. Average fisher score values of 0.765 and 0.077 were obtained for Wisconsin Diagnostic Breast Cancer dataset and Cleveland Heart Disease dataset, respectively. The use of parallel distribution mode was suggested by the authors to enhance the convergence time of the classifier.

Anter and Ali [29] developed a hybrid feature selection strategy combined with chaos theory and crow search optimization as well as fuzzy C-means algorithm. It is reported that the proposed integrated framework has the ability to reach the global optimal solution by avoiding the local optimal solution. Exploration and exploitation rates were balanced which increased the convergence speed and performance of the classifier. Experiments have been conducted for different medical datasets using different chaotic maps. For the Wisconsin Diagnostic Breast Cancer dataset, the proposed method showed an accuracy of 98.6% for the best selected attributes, whereas for Hepatitis dataset, an accuracy of 68% was obtained. The authors conducted different experiments and recorded the accuracy over different chaotic maps and evaluation criteria. Chaotic version with parallel bioinspired optimization was recommended to increase the convergence rate.

Paul and Das [30] presented an evolutionary multiobjective optimization for feature selection. In this work, a simultaneous feature selection and weighing method, instead of only feature selection, is the novelty. The authors formulated the interclass and intraclass distance measures and simultaneously used a multiobjective algorithm based on decomposition. In order to get optimal features, a penalty mechanism was introduced in the objective function, and reduced number of features are selected using a repair mechanism. Experiments were conducted for different datasets from the UCI machine learning repository and LIBSUM data repository. For Wisconsin Diagnostic Breast Cancer Dataset, it provides a better accuracy of 96.53% over the related existed methods.

Abdul Zaher and Eldeib [31] proposed a CAD system for classification of breast cancer. The authors developed the system using deep belief network and backpropagation neural network. The Liebenberg Marquardt learning function was used for the construction of backpropagation neural network. The weights are initialized using deep belief network. The experiments were conducted on Wisconsin Breast Cancer Dataset with nine features and two classes. The results show 99.68% accuracy for the Wisconsin Breast Cancer dataset. The proposed system brings an effective classification model for breast cancer. The development of parallel approach for learning such a classifier is suggested as a future work.

Christopher et al. [32] proposed a metaheuristic method called wind-driven swarm optimization for medical diagnosis. Jval, a novel evaluation metric, which considered both the accuracy of the classifier and size of the rule set, was introduced for building a classifier model. The efficiency of this work is compared with that of the traditional PSO algorithm and found to be more accurate. Experiments were carried out on clinical datasets obtained from UCI machine leaning repository, namely, Liver Disorder dataset and Cleveland Heart Disease dataset. For the liver disorder data set, the proposed method gives an accuracy of 64.60% and the heart disease data set yields 77.8% accuracy.

Aalaei et al. [33] proposed a feature selection method using genetic algorithm for breast cancer diagnosis. In this work, the authors proposed a wrapper-based approach using GA for feature selection. For classification ANN, PS classifier and GA classifier were used in this study. The idea was tested using Wisconsin Breast Cancer (WBC) dataset, Wisconsin Diagnostic Breast Cancer (WDBC) dataset, and Wisconsin Prognosis Breast Cancer (WPBC) dataset. The results from the experiments show that the proposed feature selection algorithm improves the accuracy of the classifier. The results were compared with WBC, WDBC, and WPBC datasets. The accuracy for these datasets was 96.6%, 96.6%, and 78.1%, respectively, using the GA classifier. When PS classifier was used, the accuracy for these datasets was 96.9%, 97.2%, and 78.2%, respectively. The accuracy for these datasets when ANN classifier was used was reported as 96.7%, 97.3%, and 79.2%, respectively. The accuracy of the proposed method was better compared to the existing related methods.

Christopher et al. [34] have proposed a system to predict the presence or absence of allergic rhinitis by conducting intradermal skin tests; in this work, a rule-based classification is followed. The details of skin tests conducted on different patients were collected, and different mining approaches were performed to build a Clinical Decision Support System (CDSS). A total of 872 patients were examined for this work. The CDSS diagnoses for allergic rhinitis produced an accuracy of 88.31%. This work could have been improved by introducing metaheuristic data preprocessing techniques, by using ensemble classification approaches.

Zhao et al. [35] proposed feature selection and parameter optimization for support vector machines. In this work, an approach was established with the support of genetic algorithm along with feature chromosomes, and support vector machine (SVM) was used for data classification technique. Selection of feature subset, together with setting the parameter in the SVM's training procedure, adds value to the classification accuracy. To validate the approach, experiments were conducted on the 18 datasets in UCI machine learning repository out of which Wisconsin diagnostic breast cancer belongs to the clinical domain. The results of this work are 99.00% accurate for the Wisconsin Diagnostic Breast Cancer dataset, grid search method produced an accuracy of 95.43%, and GA without the feature chromosome method produced an accuracy of 96.04%.

Zygourakis et al. [36] used a data mining algorithm called decision tree to analyse the existence of diabetes by utilizing Gini index and fuzzy decision boundary. In this work, Pima Indian Diabetes dataset from UCI machine learning repository is employed. By Preprocessing the missing value, the dataset size has been diminished to 336 instances from a total of 768 instances. In this work, three-fold cross-validation was used; the split point was estimated by implementing Gini index along with the fuzzy decision boundary. It resulted in an accuracy of 75.8% for Pima Indian Diabetes dataset.

Seera and Lim [37] proposed a model for clinical data using fuzzy min-max neural network classification and regression tree (CART) and random forest model for the hybrid intelligent system. This work proposed a system that was tested with different datasets from UCI machine learning repository, namely, liver disorder, Wisconsin diagnostic breast cancer, and Pima Indian Diabetes datasets. The proposed system was tested with three different stratified cross-validations techniques such as 2-fold, 5-fold, and 10-fold cross validations. The best performance result was achieved by applying 10-fold cross validation. The accuracies were 78.39% for Pima Indian Diabetes dataset, 95.01% for Liver Disorder dataset, and 98.84% for Wisconsin Diagnostic breast cancer dataset.

Karaolis et al. [38] developed a CAD system using decision tree algorithm to diagnose coronary heart disease. This work performed an analysis using data mining on the data collected from 1500 subjects during 2003–2006 and 2009 at Paphos General Hospital at Cyprus. C4.5 decision tree algorithm with five different splitting criteria was used to extract the rules with the following risk factors. The unchangeable risk factors considered are age, gender, family history, operations, and genetic attributes. The changeable risk factors considered are diabetes, smoking, cholesterol, hypertension, and high quantity of lipoprotein and triglycerides. This work used the splitting criteria like gain ratio, Gini index, information gain, likelihood ratio, chi-squared statistics, and distance measure. This work investigated three different models, namely, myocardial infarction (MI) vs non-MI, percutaneous coronary intervention (PCI) vs non-PCI, and coronary artery bypass graft surgery (CABG) vs non-CABG. The few important factors that were filtered by the classification rules were age, smoking, and hypertension for MI; family history, hypertension, and diabetes for PCI; and age, smoking, and history of hypertension for CABG. The classification accuracy scored by each models is MI models with 66%, PCI models with 75%, and CABG models with 75%.

Storn and Price [39] have proposed the differential evolution (DE) algorithm for optimizing nonlinear and nondifferentiable functions. The differential evolution algorithm starts with a population of candidate solutions followed by recombination, evaluation, and selection. The recombination approach deals with generating new candidate solutions based on the weighted difference between two randomly selected population solution added to a third population solution. DE was tested on standard benchmark functions, namely, Hyper–Ellipsoid function, Katsuura's function, Rastrigin's function, Griewangk's function, and Ackley's function. The DE was compared to Adaptive Simulated Annealing (ASA), the Annealed Nelder and Mead approach (ANM), the Breeder Genetic Algorithm (BGA), the EASY Evolution Strategy, and the method of Stochastic Differential Equations (SDE). In most instances, DE outperformed all of the other approaches in terms of number of function evaluations necessary to locate a global optimum of the test functions.

Yazdani and Jolai [40] have proposed a metaheuristic algorithm called Lion Optimization Algorithm (LOA) for function optimization based on the behaviour of lion troops. In Lion Optimization Algorithm (LOA), an initial population is generated by a set of randomly formed solutions called lions. Some of the lions in the initial population are selected as nomad lions and rest (resident lions) are randomly partitioned into groups known as prides, which include both male and female lions. For each lion, the best obtained solution is passed to the next iteration, and during the optimization process, the solution is updated progressively using hunting phase, moving towards the safe place phase, roaming phase, mating phase, defence phase, migration phase, Lions' Population Equilibrium phase, and convergence phase. LOA was tested on different types of benchmark functions, namely, unimodal, multimodal, hybrid, and composition. LOA achieved faster convergence and global optima achievement when compared to other metaheuristic algorithms, namely, invasive weed optimization (IWO) algorithm, biogeography-based optimization (BBO) algorithm, gravitational search algorithm (GSA), hunting search (HuS) algorithm, bat algorithm (BA), and water wave optimization (WWO) algorithm.

Krishnanand and Ghose [41] have proposed a swarm intelligence-based algorithm called Glowworm Swarm Optimization (GSO) for optimizing multimodal functions. The main objective of the method was to identify all the local optima of a function. The algorithm is modelled based on the behaviour of glowworms. GSO starts with a random population of glowworms. Each glowworm is evaluated based on the luciferin content. In each iteration, the glowworms will update their positions to increase their fitness, resulting in an optimal position. The algorithm was tested on benchmark functions, namely, Rastrigin's function, circles function, staircase function, and plateaus function. The performance of the GSO was compared with that of PSO and is found to be superior in terms of convergence speed, number of local optima captured, and computation speed.

It can be inferred from the literature that wrapper approaches which uses bioinspired algorithms for feature selection yield fruitful results. Through this work, efforts have been made to design and implement a wrapper approach for feature selection that uses three bioinspired algorithms, namely, Differential Evolution, Lion Optimization Algorithm, and Glowworm Swarm Optimization with a correlation-based ensemble feature selector.

## 3. System Framework

**Figure 1:**
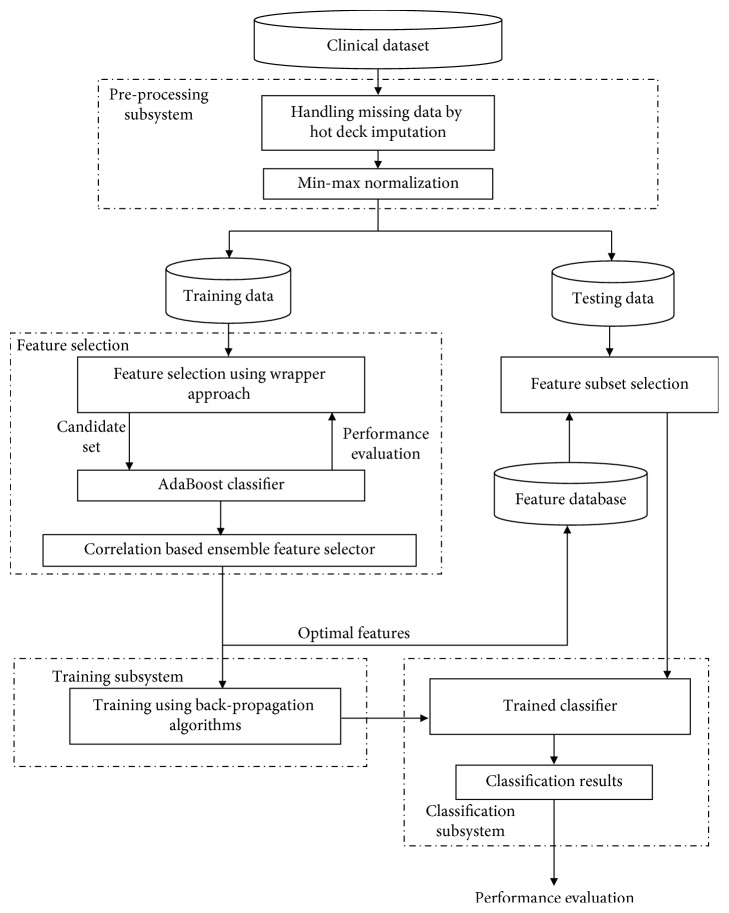
System framework.

The proposed framework consists of three subsystems, namely, preprocessing subsystem, feature selection subsystem, and classification subsystem. The preprocessing subsystem consists of missing value imputation phase and normalizing phase. The feature selection subsystem selects an optimal set of features to build the classifier model. Feature selection in this work uses the wrapper method based on the following algorithms, namely, Differential Evolution, Lion Optimization Algorithm, and Glowworm Swarm Optimization with accuracy of AdaBoostSVM as the fitness function. The classification subsystem uses Gradient Descent with momentum and Variable Learning Rate Neural Network classifier in training and testing the system. The system framework is shown in Figure 1.

### 3.1. Dataset Description

The framework was tested with two benchmark clinical datasets from UCI machine learning repository, namely, Hepatitis dataset and Wisconsin Breast Cancer (WDBC) dataset.

Hepatitis dataset consists of 155 instances with two class labels. There are 19 features in the Hepatitis dataset with 167 missing values. Outline of the attributes (features) is tabulated in Table 1. The class labels “live” and “die” from the dataset were replaced in the present work as “nonfatal” and “fatal” respectively.

Wisconsin Diagnostic Breast Cancer dataset comprises of 569 instances with 32 features and two class labels. There are no missing values in this dataset. Outline of the attributes of WDBC dataset are tabulated in Table 2.

### 3.2. Data Preprocessing

Missing or noisy values in the dataset can affect the performance of the classifier. The proposed work uses Hepatitis dataset and Wisconsin Breast Cancer dataset for experimentation among which Hepatitis dataset contains 167 missing values, whereas WDBC is free from missing or noisy values. Hot-deck imputation is used for imputing the missing values. Hot-deck imputation deals with filling in the missing values with a similar set of data from the features other than missing data field. The data are compared with the similar record, and the missing value is filled in with the value present in the similar record [42]. Since the average of missing values in Hepatitis dataset is less than 30%, missing values are imputed from a similar record that does not have a missing value.

In clinical datasets, the range and variance of one attribute may vary from another. The training data and testing data are scaled between definite limits in order to increase the efficiency of the machine learning model. This work uses a technique called min-max normalization to scale the data between 0 and 1. The min-max normalization is represented using

(1) $$\begin{array}{c} v^{\prime }=\frac{v-\min_{A}}{\max_{A}-\min_{A}}\left({\text{new}}_{\max_{A}}-{\text{new}}_{\min_{A}}\right)+{\text{new}}_{\min_{A}}, \end{array}$$

where *v*′ is the required normalized value, *v* is the current value of the variable, max_*A*_ and min_*A*_ are the maximum and minimum values of the current range, respectively, and new_max_*A*__ and new_min_*A*__ are the maximum and minimum values of the normalized range, respectively.

### 3.3. Feature Selection

The preprocessed clinical dataset is subjected to feature selection. The feature selection subsystem employs a wrapper approach using three bioinspired algorithms, namely, Differential Evolution, Lion Optimization, and Glowworm Swarm Optimization with the accuracy of AdaBoostSVM classifier as fitness function. Each bioinspired algorithm selects a subset of features yielding three feature subsets. Correlation-based ensemble feature selection is performed to select the optimal features from the three feature subsets. The reduced feature set obtained from the correlation-based ensemble feature selector is subjected to classification by a gradient-based backpropagation neural network.

#### 3.3.1. Differential Evolution

Differential Evolution (DE) is an evolutionary-based algorithm introduced by Storn and Price in 1997 [39]. DE includes mutation, crossover, and selection operations. This feature selection subsystem uses the differential evolution in a wrapper approach to select a feature subset. Accuracy of the AdaBoost with support vector machine as a base classifier is used as the fitness function. The steps involved in this process are given below.

**Step 1 .**

A Random population of 100 individuals was chosen from the dataset. The features in each individual can take a value of 0 or 1. Each individual is a possible solution which has *n* number of features.

**Step 2 .**

Each individual undergoes evaluation of fitness function using AdaBoost with support vector machine as base classifier. The accuracy of the AdaBoost classifier is taken as the fitness function.

**Step 3 .**

Genetic operations such as mutation and crossover were performed on selected individuals. First mutation operation is performed on the selected five individuals to produce offspring. Then, in crossover operation, the selected individuals are mated with the mutated individuals to produce the next generation offspring. The next generation is populated by these newly formed individuals.

**Step 4 .**

Repeat Step 2 and Step 3 until convergence is met. The convergence was met after 20 iterations. The individual having maximum fitness is taken as the feature set for further processing.

#### 3.3.2. Lion Optimization Algorithm (LOA)

Lion Optimization Algorithm is a bioinspired algorithm proposed by Maziar Yazdani in the year 2016 [40]. This feature selection subsystem uses the Lion Optimization Algorithm in a wrapper approach to select the feature subset. In LOA, an initial population is formed by a set of randomly generated solutions called lions. Some of the lions in the initial population are selected as nomad lions and rest population (resident lions) is randomly partitioned into subsets called prides. The accuracy of the AdaBoost with support vector machine as a base classifier is used as the fitness function. The steps involved in this process are given below.

**Step 5 .**

Initially a random population of 20 prides and 40 nomads was chosen from the dataset. Each pride and nomad has *n* number of features and is unisex, since both female prides and male prides go for the hunting phase regardless of its sex. The features in each individual can take a value of 0 or 1. If the feature is selected, then it is represented as 1 else 0.

**Step 6 .**

Evaluate the prides and nomads by computing the fitness value using AdaBoost with support vector machine as a base classifier.

**Step 7 .**

All pride lions in the resident territory go for hunting in a group to find their prey for food. The position of hunting lions is updated based on the following assumptions:

- These hunters have specific strategies to encircle the prey from different positions such as left, centre, and right wings positions to catch it. Hunters are divided into three subgroups based on the fitness function. Best 6 prides are taken as the centre wing, and the rest of the prides are divided for the other two wings. A dummy prey is considered in centre of hunters in the following equation:

(2) $$\begin{array}{c} \text{PREY}=\frac{{\sum \text{hunters}\left(x_{1},x_{2},x_{3},\ldots ,x_{N}\right)}^{}}{\text{number of hunters}}. \end{array}$$

(b) During the process of hunting, if the hunter improves its own fitness, the prey will escape from the hunter and find a new position using the following equation:

(3) $$\begin{array}{c} {\text{PREY}}^{\prime }=\text{PREY}+\text{rand}\left(0,1\right)*PI*\left(\text{PREY}-\text{hunter}\right), \end{array}$$

  where PREY is the current position, hunter is new position of the hunter who attacks the prey, and PI is the percentage of improvement in the fitness value of the hunter.
(c) The new positions of hunters which belong to the left and right wing are evaluated using the following equation:

(4) $$\begin{array}{c} {\text{hunter}}^{\prime }=\left\{\begin{array}{cc} \text{rand}\left(\left(2*\text{PREY}-\text{hunter}\right),\text{PREY}\right), & \left(2*\text{PREY}-\text{hunter}\right)<\text{PREY,}\\ \text{rand}\left(\text{PREY},\left(2*\text{PREY}-\text{hunter}\right)\right), & \left(2*\text{PREY}-\text{hunter}\right)>\text{PREY,} \end{array}\right. \end{array}$$

  where PREY is current position of prey, hunter is current position of hunter, and hunter′ is new position of hunter
(d) The new positions of hunters which belongs to the centre wing are evaluated using the following equation:

(5) $$\begin{array}{c} {\text{hunter}}^{\prime }=\left\{\begin{array}{cc} \text{rand}\left(\text{hunter,PREY}\right), & \text{hunter}<\text{PREY,}\\ \text{rand}\left(\text{PREY,hunter}\right), & \;\text{hunter}>\text{PREY}. \end{array}\right. \end{array}$$

**Step 8 .**

Nomad lions roam in an adaptive roaming method using equations (6) and (7):

(6) $$\begin{array}{c} {{\text{Lion}}^{\prime }}_{ij}=\left\{\begin{array}{cc} {\text{Lion}}_{ij}, & \text{if }{\text{rand}}_{j}>{\text{pr}}_{i},\\ {\text{RAND}}_{j}, & \text{otherwise,} \end{array}\right. \end{array}$$

where Lion_*i*_ is current position of *i*^th^ nomad lion, *j* is the dimension, rand_*j*_ is a uniform random number within [0, 1], RAND is random generated vector in search space, and pr_*i*_ is a probability that is calculated for each nomad lion independently:

(7) $$\begin{array}{c} {\text{pr}}_{i}=0.1+\min\;\left(0.5,\frac{\left({\text{Nomad}}_{i}-{\text{Best}}_{\text{nomad}}\right)}{{\text{Best}}_{\text{nomad}}}\right)\;,\;i=1,2,\ldots ,\text{number of nomad lions,} \end{array}$$

where Nomad_*i*_ and Best_nomad_ are cost of current position of the *i*^th^ lion in nomads and the best cost of the nomad lion, respectively.

**Step 9 .**

Since prides and nomads are considered as unisex, the mating process is done between two different lions to produce two offspring as shown in the following equations:

(8) $$\begin{array}{c} {\text{offspring}}_{j}1=\beta *{\text{Lion}}_{j}^{i}+\sum \frac{1-\beta }{\sum_{i=1}^{NR}S_{i}}*{\text{Lion}}_{j}^{k}*S_{i}, \end{array}$$

(9) $$\begin{array}{c} {\text{offspring}}_{j}2=\left(1-\beta \right)*{\text{Lion}}_{j}^{i}+\sum \frac{\beta }{\sum_{i=1}^{NR}S_{i}}*{\text{Lion}}_{j}^{k}*S_{i}, \end{array}$$

where *j* is the dimension, *S*_*i*_ equals 1 if Lions *i* and *k* are selected for mating, otherwise it equals 0, NR is the number of resident in a pride, and *β* is a randomly generated number with a normal distribution with mean value 0.5 and standard deviation 0.1.

**Step 10 .**

The accuracy of the new offspring compete with the accuracy of the prides to acquire their territory. If the new offspring is better, it replaces with the old pride and also if any nomad has higher accuracy than the pride, then it is replaced as the new pride.

**Step 11 .**

Repeat Step 2 to Step 6 for max of 100 iterations. The max fitness value pride is taken as the feature set for lion optimization algorithm.

#### 3.3.3. Glowworm Swarm Optimization

Glowworm Swarm Optimization proposed by Krishnanand and Ghose [41] is a bioinspired algorithm based on the collective behavior of glowworms. In this work, Glowworm Swarm Optimization in wrapper approach selects the feature subset. The accuracy of the AdaBoost with support vector machine as a base classifier is used as the fitness function. The steps involved in this process are given below.

**Step 12 .**

A random population of 50 glowworms is generated in the search space in such a way that each glowworm has *n* number of features. The features in each glowworm can take a value 0 or 1. If the feature is selected, then it is represented as 1 else 0. Initially, all the glowworms have equal level of luciferin *l*_0_. The constant parameters used are shown in Table 3.

**Step 13 .**

The luciferin depends on the fitness function at each glowworm position. The accuracy of the AdaBoostSVM classifier is taken as the fitness function. Each glowworm, during their luciferin update, adds to its previous luciferin level as shown in the following equation:

(10) $$\begin{array}{c} l_{i}\left(t+1\right)=\left(1-\rho \right)l_{i}\left(t\right)+\gamma J\left(x_{i}\left(t+1\right)\right), \end{array}$$

where *l*_*i*_(*t*) represents the luciferin level associated with glowworm *i* at time *t*, *ρ* is the luciferin decay constant, *γ* is the luciferin enhancement constant, and *J* (*x*_*i*_ (*t* + 1)) represents the value of the fitness function of *i*^th^ glowworm at time t

**Step 14 .**

Each *i*^th^ glowworm decides to move towards a brighter glowworm which has a greater luciferin value. Glowworm *i* selects a brighter glowworm *j* using a probabilistic mechanism as shown in the following equation:

(11) $$\begin{array}{c} p_{ij}\left(t\right)=\frac{l_{j}\left(t\right)-l_{i}\left(t\right)}{\sum_{k\varepsilon N_{i}\left(t\right)}l_{k}\left(t\right)-l_{i}\left(t\right)}, \end{array}$$

where *j εN*_*i*_(*t*), *N*_*i*_(*t*)={*j* : *d*_*ij*_(*t*) <  *r*_*d*_^*i*^(*t*); *l*_*i*_(*t*) < *l*_*j*_(*t*)} is the set of neighbors of glowworm *i* at time *t*, *d*_*ij*_(*t*) represents the Euclidean distance between the glowworms *i* and *j* at time *t*, and *r*_*d*_^*i*^(*t*) represents the variable neighborhood range associated with glowworm *i* at time *t*.

**Step 15 .**

The movement of glow worm *i* is shown in equations (12) and (13):

(12) $$\begin{array}{c} x_{i}\left(t+1\right)=x_{i}\left(t\right)+s\;\left(\frac{x_{j}\left(t\right)-x_{i}\left(t\right)}{\left|\left|x_{j}\left(t\right)-x_{i}\left(t\right)\right|\right|}\right), \end{array}$$

where *x*_*i*_(*t*) is the location of glowworm *i* at time *t*, ||*x*_*j*_(*t*) − *x*_*i*_(*t*)|| is the Euclidean distance between glowworm *i* and the glowworm *j*, and *s* is the step size.

(13) $$\begin{array}{c} r_{d}^{i}\left(t+1\right)=\min\;\left\{r_{s},\max\left\{0,\;r_{d}^{i}\left(t\right)+\beta \left(n_{t}-\left|N_{i}\left(t\right)\right|\right)\right\}\right\}, \end{array}$$

where *r*_0_ is the initial neighbourhood range of each glowworm, *β* is a constant parameter, and *n*_*t*_ is a parameter used to control the number of neighbours.

**Step 16 .**

Repeat Step 2, Step 3, and Step 4 for a max of 100 iterations. The glowworm which has the maximum luciferin is taken as the feature set for Glowworm Swarm Optimization Algorithm.

#### 3.3.4. Correlation-Based Ensemble Feature Selector

Correlation-based ensemble feature selector calculates the correlation values of each feature selected from these three bioinspired optimization approaches, and high similarity features are removed from each feature set; then, the selected features from all the three approaches are given to an ensemble feature selector. The final optimal feature set of the ensemble feature selector is obtained by majority voting on the output of their individual feature set. The steps involved in correlation-based feature selector are explained below.

**Step 17 .**

The arithmetic mode of the features selected using Differential Evolution, Lion Optimization Algorithm, and Glowworm Swarm Optimization is calculated using the following equation:

(14) $$\begin{array}{c} {\text{Out}}_{\text{ensemble feature selection}}=\text{mode}\left\{{\text{Out}}_{\text{DE}},{\text{Out}}_{\text{LION}},{\text{Out}}_{\text{GW0}}\right\}. \end{array}$$

**Step 18 .**

The correlation coefficient matrix is calculated for the features which are selected in the output of the Out ensemble feature selection using the following equation:

(15) $$\begin{array}{c} \text{correlation coefficient}=\frac{N\sum xy-\left(\sum x\right)\left(\sum y\right)}{\sqrt{\left[N\sum x^{2}-{\left(\sum x\right)}^{2}\right]\left[N\sum y^{2}-{\left(\sum y\right)}^{2}\right]}}, \end{array}$$

where *x* and *y* are attribute values under consideration and *N* is the total number of instances.

**Step 19 .**

correlation values are compared pairwise. Let *x* and *y* be the attributes which are compared in such a way that if it has correlation value greater than 0.95, *x* and *y* are highly correlated and either of them will be removed; otherwise, both will be selected by the correlation-based ensemble feature selector.

**Step 20 .**

the feature set selected by the correlation-based ensemble feature selector is given as an input to the classification subsystem

### 3.4. Classification

The neural network used in this work is a gradient descent backpropagation neural network with variable learning rates. Backpropagation neural network consists of three layers: input layer, hidden layer, and output layer. Sigmoidal function is used as the activation function for the hidden layer, and linear activation function is used for output layer. The total number of hidden nodes is calculated as in the following equation:

(16) $$\begin{array}{c} H=\left(2n+1\right), \end{array}$$

where *H* is the number of hidden nodes and *n* is the number of input nodes. The steps involved in this process are given below.

**Step 21 .**

The features selected by the correlation-based feature selector are given as the input of the BPNN. Initial parameters were initialized as shown in Table 4.

**Step 22 .**

The input of the hidden layer and the output of the hidden layer are calculated using equations (17) and (18):

(17) $$\begin{array}{c} I_{j}=\sum w_{ij}O_{j}+\emptyset_{j}, \end{array}$$

where *w*_*ij*_ are the weights of each input nodes and ∅_*j*_ is the bias.

(18) $$\begin{array}{c} O_{j}=\frac{1}{1+e^{-I_{j}}}. \end{array}$$

**Step 23 .**

The error rate is computed using gradient descent algorithm. When error rate is low, the learning rate increases, whereas when the error rate is high and the learning rate is decreased.

**Step 24 .**

The new weights and bias are updated based on the error rate and learning rate using gradient descent backpropagation algorithm. The Step 2 and Step 3 are repeated till the error rate converges.

## 4. Results and Discussion

The proposed work on Hepatitis and WDBC dataset has been implemented using Python 3.6. The feature importance of both the datasets, namely, Hepatitis and WDBC, has been calculated using information gain and is listed in Tables 5 and 6.

The proposed work selects relevant attributes using the wrapper approach based on the three bioinspired algorithms, namely, differential evolution, Lion Optimization, and Glowworm Swarm Optimization, keeping the accuracy of the AdaBoostSVM classifier as fitness function. The wrapper approach selects features which are tied to a learning algorithm and depends on the performance of the classifier. They do not depend on the values of the statistical class separability measure. The selected features using Differential Evolution, Glowworm Swarm Optimization, Lion Optimization, and Correlation-based feature selector for both datasets are shown in Tables 7 and 8.

Feature selection plays a major role in healthcare applications for efficient classification [43–47]. Devijver and Kittler define feature selection as the process of extracting the relevant information from the raw data to improve the classification performance [48]. Feature selection gives a clear view of data visualization and data understanding to improve the prediction performance [49].

In the case of Hepatitis dataset, out of 18 attributes, 3 attributes, namely, Anorexia, Liver_Big, and Spleen_Palpable are pruned, and all others are selected by the proposed correlation-based feature selector, whereas in the case of WDBC, out of 31 attributes, 12 attributes, namely, P_id, Mean_perimeter, Standard_error_perimeter, Standard_error_area, Standard_error_smoothness, Standard_error_concavity, Concavepoints_standard_error, Standard_error_symmetry, Standard_error_fractaldimension, Worst_radius, Worst_perimeter, and Worst_area are pruned, and all others are selected by the proposed correlation-based feature selector. Also, the authors have consulted with clinicians and research papers for the medical relevance of the selected features [50–53].

Accuracy, precision, sensitivity, and specificity are used to assess the performance of classifiers which are represented using equations (19)–(22):

(19) $$\begin{array}{c} \text{accuracy}=\frac{\text{samples correctly classified }}{\text{total samples classified}}=\frac{\text{TP}+\text{TN}}{\text{TP}+\text{FP}+\text{FN}+\text{TN}}, \end{array}$$

(20) $$\begin{array}{c} \text{precision}=\frac{\text{samples correctly classified as positives }}{\text{total samples classified as positives}}=\frac{\text{TP}}{\text{TP}+\text{FP}}, \end{array}$$

(21) $$\begin{array}{c} \text{sensitivity}=\frac{\text{samples correctly classified as postives}}{\text{total postives samples in the test dataset}}=\frac{\text{TP}}{\text{TP}+\text{FN}}, \end{array}$$

(22) $$\begin{array}{c} \text{specificity}=\frac{\text{samples correctly classified as negatives}}{\text{total negatives samples in the test dataset}}=\frac{\text{TN}}{\text{TN}+\text{FP}}, \end{array}$$

where TP, TN, FP, and FN are true-positive rate, true-negative rate, false-positive rate, and false-negative rate, respectively, which are obtained from the confusion matrix.

**Figure 2:**
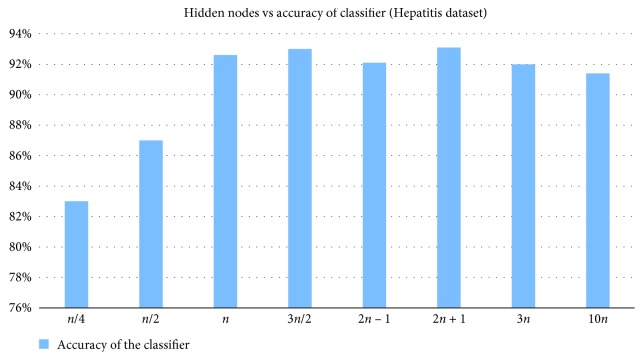
Comparison of classifier accuracy achieved by changing the number of hidden nodes for Hepatitis dataset.

**Figure 3:**
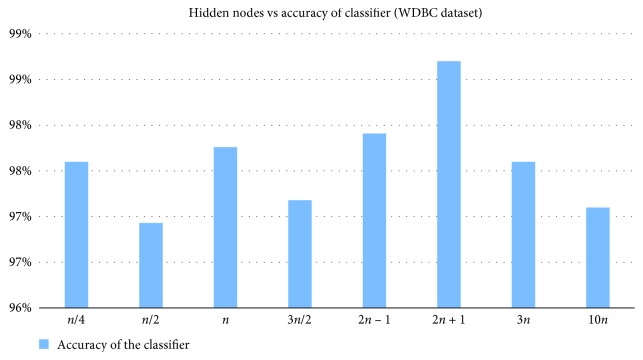
Comparison of classifier accuracy achieved by changing the number of hidden nodes for WDBC dataset.

The classifier accuracy is compared by changing the hidden nodes. From Figures 2 and 3, it can be inferred that the BPNN experimented with (2*n* + 1) hidden nodes has yielded better results for both Hepatitis and WDBC datasets.

The confusion matrix of the BPNN classifier with (2*n* + 1) hidden nodes for the datasets hepatitis and WDBC is shown in Tables 9 and 10. For Hepatitis dataset, there are 38 true negatives, 39 true positives, 2 false positives, and 3 false negatives, whereas for WDBC, there are 118 true negatives, 116 true positives, 2 false positives, and 1 false negative.

Table 11 indicates that the proposed framework has achieved an accuracy of 98.734%, precision of 98.305%, sensitivity of 99.145%, and specificity of 98.333% for WDBC and an accuracy of 93.902%, precision of 95.121%, sensitivity of 92.857%, and specificity of 95% for hepatitis. The results obtained were validated by physicians.

The performance of correlation-based ensemble feature selector was compared with results of individual feature selection algorithms (Differential Evolution, Glowworm Swarm Optimization, and Lion Optimization Algorithm) as shown in the Tables 12 and 13 for Hepatitis and WDBC datasets. It is observed that the performance of correlation-based ensemble feature selection with backpropagation neural network classifier outperforms the other single optimization algorithms (Differential Evolution, Glowworm Swarm Optimization, and Lion Optimization Algorithm) with backpropagation neural network for the WDBC and Hepatitis datasets.

The performance of the proposed framework was also compared with results of other classifiers (naive Bayes, J48, decision table, AdaBoostMI, multilayer perceptron, and random forest) using the WEKA tool, and the results are tabulated in Tables 14, and 15 for WDBC and Hepatitis datasets. It is observed that the performance of correlation-based ensemble feature selection with backpropagation neural network classifier outperforms the other classifiers for the WDBC and Hepatitis datasets.

## 5. Conclusion and Future Work

This work presents a novel feature selection strategy which uses a wrapper approach comprising of three bioinspired algorithms, namely, Differential Evolution, Lion Optimization Algorithm, and Glowworm Swarm Optimization Algorithm with AdaBoostSVM as the underlying classifier. A correlation-based ensemble feature selector is used to select the relevant features from the clinical dataset. The novelty of correlation-based ensemble feature selection attributes to the diverse bioinspired algorithms used to evaluate the features. The system has achieved an accuracy of 93.902%, sensitivity of 92.857%, specificity of 95%, and precision of 95.121% for hepatitis and an accuracy of 98.734%, sensitivity of 99.145%, specificity of 98.333%, and precision of 98.305% for WDBC. The proposed framework can be tailored to develop CDSS for other clinical datasets with domain specific changes. Other bioinspired algorithms and classifiers can also be used to enhance the performance of the proposed framework.

## Figures and Tables

**Table 1:** Outline of hepatitis datasets.

S. no.	Feature	Description	Datatype
1.	Age	Age of the patient	Numerical
2	Sex	Gender of the patient	Categorical
3	Steroid	Whether the patient has taken anabolic steroids or not	Boolean
4	Antivirals	Whether the patient has taken antivirals or not	Boolean
5	Fatigue	Whether the patient has experienced extreme tiredness or not	Boolean
6	Malaise	Whether the patient is having a vague feeling of body discomfort	Boolean
7	Anorexia	Whether the patient has lack or loss of appetite for food	Boolean
8	Liver big	Whether the patient's liver is enlarged or not	Boolean
9	Liver firm	Whether the patient's liver is firm or not	Boolean
10	Spleen palpable	Whether the patient's spleen is enlarged or not	Boolean
11	Spiders	Whether the blood vessels are near the skin surface due to the increased estrogen level.	Boolean
12	Ascites	Whether the fluid is accumulated in the peritoneal cavity or not	Boolean
13	Varices	Whether the patient is having bleeding from varices or not	Boolean
14	Bilirubin	The amount of bilirubin in the blood sample	Numerical
15	Alk phosphate	Level of alkane phosphate in the blood sample	Numerical
16	Sgot	The amount of serum lutamic oxalo acetic transaminase in the blood	Numerical
17	Albumin	The amount of serum albumin protein in the clear liquid portion of the blood sample	Numerical
18	Protime	Time taken for blood plasma to clot	Numerical
19	Histology	Class attribute indicates whether the patient survives or not	Boolean

**Table 2:** Outline of WDBC dataset.

S. no	Feature	Description	Data type
1	ID	Patient identification number	Numerical
2	Diagnosis	Malignant or benign	Character

3	Radius (mean)	Mean of distances from centre to points on the perimeter	Real
4	Radius (error)		Real
5	Radius (worst)		Real

6	Texture (mean)	Standard deviation of grey scale values	Real
7	Texture (error)		Real
8	Texture (worst)		Real

9	Perimeter (mean)	Perimeter of cell nucleus	Real
10	Perimeter (error)		Real
11	Perimeter (worst)		Real

12	Area (mean)	Area of cell	Real
13	Area (error)		Real
14	Area (worst)		Real

15	Smoothness (mean)	Local variation in radius lengths	Real
16	Smoothness (error)		Real
17	Smoothness (worst)		Real

18	Compactness (mean)	(perimeter^2/area—1.0)	Real
19	Compactness (error)		Real
20	Compactness (worst)		Real

21	Concavity (mean)	Severity of concave portions of the contour	Real
22	Concavity (error)		Real
23	Concavity (worst)		Real

24	Concave (mean)	Number of concave portions of the contour	Real
25	Concave (error)		Real
26	Concave (worst)		Real

27	Symmetry (mean)	Measure of cell symmetry	Real
28	Symmetry (error)		Real
29	Symmetry (worst)		Real

30	Fractal dimension (mean)	(“Coastline approximation”—1)	Real
31	Fractal dimension (error)		Real
32	Fractal dimension (worst)		Real

**Table 3:** Parameter setting for Glowworm Swarm Optimization.

Parameter	Value
*ρ*	0.4
*γ*	0.6
*β*	0.08
*n*_*t*_	5
*s*	0.03
*l*_0_	5

**Table 4:** Parameter setting for BPNN.

Parameter	Value	Meaning
*n*	Features selected by correlation-based ensemble feature selector	Number of input nodes
*H*	(2*n* + 1)	Number of hidden nodes
*H*_layer_	1	Hidden layer
*O*	Linear	Output

Initial weights and bias are randomly assigned with small random variables ranging from −0.5 to 0.5, and the learning rate is kept as 0.5.

**Table 5:** Feature importance of Hepatitis dataset.

S. no.	Feature	Feature importance	Rank
1	Age	0.335503	3
2	Sex	0.014356	15
3	Steroid	0.011443	16
4	Antivirals	0.033793	11
5	Fatigue	0.022269	13
6	Malaise	0.019788	14
7	Anorexia	0.010061	17
8	Liver big	0.007907	18
9	Liver firm	0.032248	12
10	Spleen palpable	0.036895	10
11	Spiders	0.095853	9
12	Ascites:	0.099008	8
13	Varices	0.110238	7
14	Bilirubin	0.202373	6
15	Alk phosphate	0.532782	1
16	SGOT	0.511008	2
17	Albumin	0.21938	5
18	Protime	0.294931	4

**Table 6:** Feature importance of WDBC dataset.

S. no.	Feature	Feature importance	Rank
1	ID	0.852635	24
2	Radius (mean)	0.860782	22
3	Radius (error)	0.835712	27
4	Radius (worst)	0.926704	10
5	Texture (mean)	0.928031	8
6	Texture (error)	0.776179	29
7	Texture (worst)	0.909129	16
8	Perimeter (mean)	0.93506	2
9	Perimeter (error)	0.94209	1
10	Perimeter (worst)	0.735037	30
11	Area (mean)	0.836177	26
12	Area (error)	0.933734	5
13	Area (worst)	0.864297	20
14	Smoothness (mean)	0.931545	6
15	Smoothness (error)	0.925377	11
16	Smoothness (worst)	0.93505	3
17	Compactness (mean)	0.923189	12
18	Compactness (error)	0.928030	9
19	Compactness (worst)	0.858593	23
20	Concavity (mean)	0.818137	28
21	Concavity (error)	0.917486	14
22	Concavity (worst)	0.900307	17
23	Concave (mean)	0.863435	21
24	Concave (error)	0.898584	18
25	Concave (worst)	0.935045	4
26	Symmetry (mean)	0.719719	31
27	Symmetry (error)	0.918347	13
28	Symmetry (worst)	0.930219	7
29	Fractal dimension (mean)	0.914832	15
30	Fractal dimension (error)	0.845395	25
31	Fractal dimension (worst)	0.891554	19

**Table 7:** Features selected for hepatitis dataset.

	Age	Sex	Steroid	Antivirals	Fatigue	Malaise	Anorexia	Liver_big	Liver_firm	Spleen_palpable	Spiders	Ascites	Varices	Bilirubin	Alk_phosphate	Sgot	Albumin	Histology	Class
DE	1	0	1	1	0	1	1	1	1	0	1	1	1	1	1	1	0	0	1
GSO	1	1	0	1	1	1	0	0	1	0	1	1	0	0	1	0	1	1	1
LION	1	1	1	1	1	1	0	0	1	1	1	1	1	1	1	1	1	1	1
*Correlation-based feature selector*	1	1	1	1	1	1	0	0	1	0	1	1	1	1	1	1	1	1	1

**Table 8:** Features selected for WDBC dataset.

Correlation-based feature selector	LION	GSO	DE	
0	0	0	0	P_id
1	1	1	0	Mean_radius
1	1	1	1	Mean_texture
0	1	1	0	Mean_perimeter
1	1	1	1	Mean_area
1	1	1	0	Mean_smoothness
1	1	1	0	Mean_compactness
1	1	1	0	Mean_concavity
1	1	1	1	Concavepoints_mean
1	1	1	1	Mean_symmetry
1	1	1	0	Mean_fractaldimension
1	1	1	1	Standard_error_radius
1	1	0	1	Standard_error_texture
0	1	1	0	Standard_error_perimeter
0	0	0	0	Standard_error_area
0	0	0	1	Standard_error_smoothness
1	1	1	0	Standard_error_compactness
0	0	0	0	Standard_error_concavity
0	0	0	1	Concavepoints_standard_error
0	1	0	0	Standard_error_symmetry
0	0	0	1	Standard_error_fractaldimension
0	0	0	1	Worst_radius
1	1	1	0	Worst_texture
0	0	0	1	Worst_perimeter
0	1	1	1	Worst_area
1	1	1	1	Worst_smoothness
1	0	1	1	Worst_compactness
1	1	0	1	Worst_concavity
1	1	0	1	Concavepoints_worst
1	1	1	0	Worst_symmetry
1	0	1	1	Worst_fractaldimension
1	1	1	1	Diagnosis

**Table 9:** Confusion matrix for proposed framework used to train and test the Hepatitis dataset.

	Predicted		
Expected		Nonfatal	Fatal
	Nonfatal	38 (TN)	2 (FP)
	Fatal	3 (FN)	39 (TP)

**Table 10:** Confusion matrix for proposed framework used to train and test the WDBC dataset.

	Predicted		
Expected		Benign	Malignant
	Benign	118 (TN)	2 (FP)
	Malignant	1 (FN)	116 (TP)

**Table 11:** Performance evaluation of the proposed framework.

Measure	WDBC (%)	Hepatitis (%)
Accuracy	98.734	93.902
Precision	98.305	95.121
Sensitivity	99.145	92.857
Specificity	98.333	95

**Table 12:** Performance of correlation-based ensemble feature selector and individual feature selector for Hepatitis dataset.

Measure	Proposed work (%)	DE (%)	GSO (%)	Lion (%)
Accuracy	93.902	91.46	92.6	92.68
Precision	95.121	92.68	95	95.12
Sensitivity	92.857	90.69	90.47	90.69
Specificity	95	92.5	95	94.87

**Table 13:** Performance of correlation-based ensemble feature selector and individual feature selector for WDBC dataset.

Measure	Proposed work (%)	DE (%)	GSO (%)	Lion (%)
Accuracy	98.734	97.03	97.45	97.45
Precision	98.305	95.86	96.66	95.90
Sensitivity	99.145	98.30	98.30	99.15
Specificity	98.333	95.76	96.61	95.76

**Table 14:** Performance comparison of proposed work with other classifiers for Hepatitis dataset.

Measure	Naive Bayes	J48	Decision table	AdaBoostMI	Multilayer Perceptron	Random forest	Proposed work
Accuracy	0.8387	0.8064	0.7806	0.8064	0.8452	0.8323	0.93902
Precision	0.8450	0.7980	0.7810	0.7980	0.8390	0.8250	0.95121
Sensitivity	0.8390	0.8060	0.7810	0.9417	0.8450	0.8819	0.92857
Specificity	0.9083	0.8661	0.8618	0.8661	0.8898	0.8320	0.95

**Table 15:** Performance comparison of proposed work with other classifiers for WDBC dataset.

Measure	Naive Bayes	J48	Decision table	AdaBoostMI	Multilayer perceptron	Random forest	Proposed work
Accuracy	0.9297	0.9312	0.9350	0.9472	0.9630	0.9666	0.98734
Precision	0.9300	0.9320	0.9350	0.9470	0.9630	0.9670	0.98305
Sensitivity	0.9300	0.9310	0.9350	0.9417	0.9630	0.9670	0.99145
Specificity	0.8716	0.8950	0.9268	0.9470	0.9569	0.9707	0.98333

## Data Availability

The data supporting this study are from previously reported studies and datasets, which have been cited. The datasets used in this research work are available at UCI Machine Learning repository.

## References

[1] Han J., Kamber M., Pei J. (2011). *Data Mining Concepts and Techniques*.

[2] Bellazzi R., Zupan B. (2008). Predictive data mining in clinical medicine: current issues and guidelines. *International Journal of Medical Informatics*.

[3] Nahato K. B., Harichandran K. N., Arputharaj K. (2015). Knowledge mining from clinical datasets using rough sets and backpropagation neural network. *Computational and Mathematical Methods in Medicine*.

[4] Christopher J. J., Nehemiah H. K., Arputharaj K., Moses G. L. (2016). Computer-assisted medical decision-making system for diagnosis of Urticaria. *MDM Policy & Practice*.

[5] Vijaya K., Nehemiah H. K., Kannan A., Bhuvaneswari N. G. (2010). Fuzzy neuro genetic approach for predicting the risk of cardiovascular diseases. *International Journal of Data Mining, Modelling and Management*.

[6] Elizabeth D. S., Raj C. S. R., Nehemiah H. K., Kannan A. (2012). Computer-aided diagnosis of lung cancer based on analysis of the significant slice of chest computed tomography image. *IET Image Processing*.

[7] Elizabeth D. S., Nehemiah H. K., Raj C. S. R., Kannan A. (2012). A novel segmentation approach for improving diagnostic accuracy of CAD systems for detecting lung cancer from chest computed tomography images. *Journal of Data and Information Quality (JDIQ)*.

[8] Elizabeth D. S., Kannan A., Nehemiah H. K. (2009). Computer-aided diagnosis system for the detection of bronchiectasis in chest computed tomography images. *International Journal of Imaging Systems and Technology*.

[9] Darmanayagam S. E., Harichandran K. N., Cyril S. R. R., Arputharaj K. (2013). A novel supervised approach for segmentation of lung parenchyma from chest CT for computer-aided diagnosis. *Journal of Digital Imaging*.

[10] Sweetlin J. D., Nehemiah H. K., Kannan A. (2018). Computer aided diagnosis of pulmonary hamartoma from CT scan images using ant colony optimization based feature selection. *Alexandria Engineering Journal*.

[11] Raj R., Nehemiah H. K., Elizabeth D. S., Kannan A. (2018). A novel feature-significance based k-nearest neighbour classification approach for computer aided diagnosis of lung disorders. *Current Medical Imaging Reviews*.

[12] Titus A., Nehemiah H. K., Kannan A. (2015). Classification of interstitial lung diseases using particle swarm optimized support vector machine. *International Journal of Soft Computing*.

[13] Jane Y. N., Nehemiah H. K., Arputharaj K. (2016). A Q-backpropagated time delay neural network for diagnosing severity of gait disturbances in Parkinson’s disease. *Journal of Biomedical Informatics*.

[14] Nancy J. Y., Khanna N. H., Kannan A. (2017). A bio-statistical mining approach for classifying multivariate clinical time series data observed at irregular intervals. *Expert Systems with Applications*.

[15] Leema N., Khanna Nehemiah H., Kannan A., Jabez Christopher J. (2016). Computer aided diagnosis system for clinical decision making: experimentation using Pima Indian diabetes dataset. *Asian Journal of Information Technology*.

[16] Song L., Smola A., Gretton A., Borgwardt K. M., Bedo J. Supervised feature selection via dependence estimation.

[17] Dy J. G., Brodley C. E. (2004). Feature selection for unsupervised learning. *Journal of Machine Learning Research*.

[18] Xu Z., King I., Lyu M. R. T., Jin R. (2010). Discriminative semi-supervised feature selection via manifold regularization. *IEEE Transactions on Neural Networks*.

[19] Liu H., Yu L. (2005). Toward integrating feature selection algorithms for classification and clustering. *IEEE Transactions on Knowledge and Data Engineering*.

[20] Zhao Z., Liu H. Semi-supervised feature selection via spectral analysis.

[21] Reddy M. J., Kumar D. N. (2012). Computational algorithms inspired by biological processes and evolution. *Current Science*.

[22] Kar A. K. (2016). Bio inspired computing—a review of algorithms and scope of applications. *Expert Systems with Applications*.

[23] Leema N., Nehemiah H. K., Kannan A. (2016). Neural network classifier optimization using differential evolution with global information and back propagation algorithm for clinical datasets. *Applied Soft Computing*.

[24] Sweetlin J. D., Nehemiah H. K., Kannan A. (2017). Feature selection using ant colony optimization with tandem-run recruitment to diagnose bronchitis from CT scan images. *Computer Methods and Programs in Biomedicine*.

[25] Emary E., Zawbaa H. M., Hassanien A. E. (2016). Binary grey wolf optimization approaches for feature selection. *Neurocomputing*.

[26] Nahato K. B., Nehemiah K. H., Kannan A. (2016). Hybrid approach using fuzzy sets and extreme learning machine for classifying clinical datasets. *Informatics in Medicine Unlocked*.

[27] Mafarja M., Eleyan D., Abdullah S., Mirjalili S. S-shaped vs. V-shaped transfer functions for ant lion optimization algorithm in feature selection problem.

[28] Zawbaa H. M., Emary E., Grosan C., Snasel V. (2018). Large-dimensionality small-instance set feature selection: a hybrid bio-inspired heuristic approach. *Swarm and Evolutionary Computation*.

[29] Anter A. M., Ali M. (2019). Feature selection strategy based on hybrid crow search optimization algorithm integrated with chaos theory and fuzzy c-means algorithm for medical diagnosis problems. *Soft Computing*.

[30] Paul S., Das S. (2015). Simultaneous feature selection and weighting - an evolutionary multi-objective optimization approach. *Pattern Recognition Letters*.

[31] Abdel-Zaher A. M., Eldeib A. M. (2016). Breast cancer classification using deep belief networks. *Expert Systems with Applications*.

[32] Christopher J. J., Nehemiah H. K., Kannan A. (2015). A swarm optimization approach for clinical knowledge mining. *Computer Methods and Programs in Biomedicine*.

[33] Aalaei S., Shahraki H., Rowhanimanesh A., Eslami S. (2016). Feature selection using genetic algorithm for breast cancer diagnosis: experiment on three different datasets. *Iranian Journal of Basic Medical Sciences*.

[34] Christopher J. J., Nehemiah H. K., Kannan A. (2015). A clinical decision support system for diagnosis of allergic rhinitis based on intradermal skin tests. *Computers in Biology and Medicine*.

[35] Zhao M., Fu C., Ji L., Tang K., Zhou M. (2011). Feature selection and parameter optimization for support vector machines: a new approach based on genetic algorithm with feature chromosomes. *Expert Systems with Applications*.

[36] Zygourakis C. C., Oh T., Sun M. Z., Barani I., Kahn J. G., Parsa A. T. (2014). Surgery is cost-effective treatment for young patients with vestibular schwannomas: decision tree modeling of surgery, radiation, and observation. *Neurosurgical Focus*.

[37] Seera M., Lim C. P. (2014). A hybrid intelligent system for medical data classification. *Expert Systems with Applications*.

[38] Karaolis M. A., Moutiris J. A., Hadjipanayi D., Pattichis C. S. (2010). Assessment of the risk factors of coronary heart events based on data mining with decision trees. *IEEE Transactions on Information Technology in Biomedicine*.

[39] Storn R., Price K. (1997). Differential evolution–a simple and efficient heuristic for global optimization over continuous spaces. *Journal of Global Optimization*.

[40] Yazdani M., Jolai F. (2016). Lion optimization algorithm (LOA): a nature-inspired metaheuristic algorithm. *Journal of Computational Design and Engineering*.

[41] Krishnanand K. N., Ghose D. (2009). Glowworm swarm optimisation: a new method for optimising multi-modal functions. *International Journal of Computational Intelligence Studies*.

[42] Andridge R. R., Little R. J. A. (2010). A review of hot deck imputation for survey non-response. *International Statistical Review*.

[43] Ghazavi S. N., Liao T. W. (2008). Medical data mining by fuzzy modeling with selected features. *Artificial Intelligence in Medicine*.

[44] Lee S.-K., Chung P.-C., Chang C.-I. (2003). Classification of clustered microcalcifications using a Shape Cognitron neural network. *Neural Networks*.

[45] López Y., Novoa A., Guevara M. A., Silva A. (2007). Breast cancer diagnosis based on a suitable combination of deformable models and artificial neural networks techniques. *Iberoamerican Congress on Pattern Recognition*.

[46] Soltanian-Zadeh H., Rafiee-Rad F., Pourabdollah-Nejad D S. (2004). Comparison of multiwavelet, wavelet, Haralick, and shape features for microcalcification classification in mammograms. *Pattern Recognition*.

[47] Wei J., Sahiner B., Hadjiiski L. M. (2005). Computer-aided detection of breast masses on full field digital mammograms. *Medical Physics*.

[48] Devijver P. A., Kittler J. (1982). *Pattern Recognition: A Statistical Approach*.

[49] Guyon I., Elisseeff A. (2003). An introduction to variable and feature selection. *Journal of Machine Learning Research*.

[50] Štimac D., Milic S., Dintinjana R. D., Kovac D., Ristic S. (2002). Androgenic/Anabolic steroid-induced toxic hepatitis. *Journal of Clinical Gastroenterology*.

[51] Cohen J. A., Kaplan M. M. (1979). The SGOT/SGPT ratio? An indicator of alcoholic liver disease. *Digestive Diseases and Sciences*.

[52] Scutt D., Manning J. T., Whitehouse G. H., Leinster S. J., Massey C. P. (1997). The relationship between breast asymmetry, breast size and the occurrence of breast cancer. *The British Journal of Radiology*.

[53] Karahaliou A. N., Boniatis I. S., Skiadopoulos S. G. (2008). Breast cancer diagnosis: analyzing texture of tissue surrounding microcalcifications. *IEEE Transactions on Information Technology in Biomedicine*.

